# Morphological Observations and Fatty Acid Composition of Indoor-Cultivated *Cordyceps sinensis* at a High-Altitude Laboratory on Sejila Mountain, Tibet

**DOI:** 10.1371/journal.pone.0126095

**Published:** 2015-05-04

**Authors:** Lian-Xian Guo, Xiao-Ming Xu, Fu-Rui Liang, Jian-Ping Yuan, Juan Peng, Chou-Fei Wu, Jiang-Hai Wang

**Affiliations:** 1 School of Public Health, Guangdong Medical College, Dongguan, Guangdong, People’s Republic of China; 2 Guangdong Provincial Key Laboratory of Marine Resources and Coastal Engineering, Sun Yat-Sen University, Guangzhou, Guangdong, People’s Republic of China; Leibniz-Institute of Vegetable and Ornamental Crops, GERMANY

## Abstract

*Cordyceps sinensis*, a caterpillar entomopathogenic fungus-host larva complex, is a rare medicinal herb found in the Qinghai-Tibetan Plateau and its surrounding high-altitude areas. The alternation of generations in the life cycle, whatever the fungus or its host insect, requires special growth conditions. However, it is difficult to simulate the growth conditions of *C*. *sinensis*, which hinders its artificial cultivation. In this work, the life cycle from the host larva to *C*. *sinensis *was observed in an indoor-cultivation laboratory at 4,200 m a.s.l. on Sejila Mountain, Tibet. Comparative examinations between indoor-cultivated and wild *C*. *sinensis *demonstrated that the indoor-cultivated *C*. *sinensis *preferred to germinate multiple long, slim stromata at diverse positions on dead larvae, including but not limited to their heads. Their fatty acid composition shows a significant difference in the levels of polyunsaturated fatty acids (PUFAs). In indoor-cultivated *C*. *sinensis*, PUFAs constituted 24.59% and 49.43%, respectively, of neutral and polar lipids; meanwhile, in wild *C*. *sinensis*, PUFAs represented 34.34% and 61.25% of neutral and polar lipids, respectively. These observations and fatty acid data suggest that environmental factors, particularly temperature, soil pressure and light intensity, strongly affect the growth of *C*. *sinensis*. Our new findings may provide important information for improving techniques for the large-scale artificial cultivation of *C*. *sinensis*.

## Introduction

*Cordyceps sinensis* [1] is a well-known fungus-larva symbiote that is naturally distributed in the Qinghai-Tibetan Plateau and the adjacent high-altitude areas [2]. (Note: The Latin name *Cordyceps sinensis* (Berk.) *Sacc*. is used for both the fungus and the wild fungus-caterpillar complex product indiscriminately. The fungus has been re-named *Ophiocordyceps sinensis* (Berk.) Sung et al.; however, the Latin name for the wild product has remained unchanged. In this paper, we use the term *O*. *sinensis* to refer to the fungus/fungi and the name *C*. *sinensis* to refer to the fungus-caterpillar complex.) [3] The life cycle of *O*. *sinensis* includes two stages: anamorphic (asexual) and teleomorphic (sexual). During the asexual stage, the hyphae or asexual conidia of *Hirsutella sinensis* [4] infect their host larva, which lives in topsoils and belongs to the genus *Thitarodes* (Lepidoptera: Hepialidae) [5,6]. Fungal infection may induce a larva to sluggishly crawl upwards at a position near the ground. The fungus multiplies in the body cavity of the host larva, and it may ultimately fill the hemocoelom with its threadlike hyphae. The host larva gradually becomes hard and turns into a caterpillar-shaped sclerotium (“stiff worm”). After overwintering, the fungal fruiting body, by using the dead host larva as a substrate, ruptures the larval cuticle, generally at the head, and its stroma emerges from the topmost soil layer in late spring. The stroma continually grows upwards, breaks through soils, and finally forms a stalked fruiting body with the ability to produce multiseptate, elongate-fusoid and non-fragmenting ascospores (sexual stage). Ascospores released from the perithecia may infect new larvae [7].

*C*. *sinensis* has been used in China for more than 2,000 years as a rare health food and a traditional medicinal herb [8] to treat diverse chronic diseases. It exhibits beneficial effects on renal and hepatic functions and an immunomodulation-related anti-tumor activity [9]. Modern pharmacological studies have shown that *C*. *sinensis* possesses many chemical constituents [10] with specific pharmacological activities [11], which have recently attracted much attention [12]. The pronounced medicinal function has resulted in a large demand for wild *C*. *sinensis*. However, the population of wild *C*. *sinensis* is extremely limited due to its complicated life cycle, obligate parasitism [13,14] and ecogeographical preferences [2,15]. Furthermore, excessive excavation, human destruction of its habitats and the upward movement of the snow line due to global climate change [16] have further aggravated the decrease in the yield of *C*. *sinensis* in the past twenty years, and the retail price of wild *C*. *sinensis* products has risen correspondingly quickly [17,18]. Most scientists working to alleviate the lack of wild *C*. *sinensis* have focused on the cultivation and fermentation of *Cordyceps* mycelium, but a few have attempted artificial cultivation and have succeeded in cultivating *C*. *sinensis* in the laboratory according to the following procedures: feeding wild larvae indoors and cultivating *C*. *sinensis* indoors or outdoors after manually infecting larvae with conidia or ascospores [19]. However, because the fungus and its host insect exhibit an alternation of generations in the life cycle, and each generation requires its specific growth conditions, the large-scale artificial cultivation of *C*. *sinensis* has not yet been achieved. To achieve artificial cultivation on a large scale, our group studied the diet of the host *T*. larva of *O*. *sinensis* and revealed that the soil humus in its habitats was a potential food source for outdoor breeding of the host larvae on a large scale [20]. We further investigated the fatty acid composition of neutral and polar lipids from wild *C*. *sinensis* produced in several high-altitude areas [15] in comparison to that of *Cordyceps militaris* [21] for determining the key factors that affected the growth of *C*. *sinensis*. The results suggested that the evident differences in the degree of polar lipid unsaturation might both be inherited from the special fatty acid composition in the host *T*. larva as its substrate and be subsequently biosynthesized de novo by the fungus in response to environmental factors [15,21], especially low temperatures that might lower the fluidity of the cell membrane. Therefore, the exhaustive simulation of main environmental factors by reference to the natural habitat of *C*. *sinensis* [22] is critical for its artificial cultivation on a large scale. In this study, the wild larvae collected from the habitat were reared in the laboratory, and a subset of the larvae could spontaneously transform into *C*. *sinensis*. This type of *C*. *sinensis* was designated indoor *C*. *sinensis*, which was neither totally wild *C*. *sinensis* produced in natural habitats nor the fully artificially cultivated *C*. *sinensis* from previous reports [19]. Here, we present the successful indoor cultivation of *C*. *sinensis* at a high-altitude laboratory on Sejila Mountain for the first time, and we examine the relationship between the wild and indoor-cultivated *C*. *sinensis*, with special reference to the differences in their morphological characters and in their fatty acid compositions of neutral/polar lipids. Several key environmental factors affecting the growth and appearance of *C*. *sinensis*, including temperature, luminous intensity and soil pressure are also discussed on the basis of the new observations. The results may provide important information to improve the techniques for the large-scale artificial cultivation of *C*. *sinensis*.

## Materials and Methods

### Sampling

Wild host *T*. larvae for this study were randomly collected in a shrubbery area (longitude: 94°36’16 E; latitude: 29°36’20 N; altitude: 4,200 m) in late June 2007 on Sejila Mountain, Tibet, which is one of the best known habitats of *C*. *sinensis*. Field observations indicated that wild *T*. larvae lived in humus-layer soil tunnels at 920 cm below the surface, and the population density ranged from 13 to 40 larvae per square meter in area. These larvae were temporarily fed in a ventilated box with soil from the natural habitat spread on its floor, and they were delivered to a laboratory at 4,200 m elevation within 2 h.

### Indoor cultivation of *C*. *sinensis*

In the laboratory, *ca*. 15–20 larvae at the 4th and 5th instars were raised in each polyurethane foam box [60 cm (length) × 40 cm (width) × 30 cm (height); 20 boxes in total], which was floored with the habitat mosses (35 cm thick). The larvae were fed with clean, fresh carrot slices of 0.5 to 1 cm in thickness. The indoor temperature was maintained at 8–12°C during breeding.

During the breeding cycle, some of the *T*. larvae died, due to diverse reasons. The dead larvae were promptly separated from the healthy ones, and the dead larvae were placed in an incubator with habitat mosses spread on its floor. Temperature and relative humidity in the incubator were maintained at −4°C to 4°C and 70% to 90%, respectively. Some of the dead larvae gradually transformed into *C*. *sinensis*, referred to herein as indoor *C*. *sinensis*.

### Analysis of fatty acid composition

#### Chemicals and reagents

The solvents used in this study, including petroleum ether (PE; boiling point, 30–60°C), tetrahydrofuran (THF), dichloromethane, ethyl acetate, acetic acid, and anhydrous methanol, and the reagents, including anhydrous sodium sulfate, sodium carbonate and sulfuric acid (Guangzhou Chemical Reagent Factory, Guangzhou), were analytical grade. PE was purified by three sequential washes with concentrated sulfuric acid, 2% sodium carbonate solution and water, followed by drying over anhydrous sodium sulfate and distillation using a rectifying column (Shuniu 1081, Shubo, Chengdu). THF, dichloromethane and ethyl acetate were purified using distillation with a rectifying column. Anhydrous PE, methanol (HPLC grade, Merck, Darmstadt) and THF were obtained by refluxing the solvents with sodium wire, followed by redistillation. Sodium methoxide (5%) was prepared by the addition of a calculated amount of fresh sodium wire to anhydrous methanol.

#### Extraction of lipids and transesterification

Thirteen samples of indoor *C*. *sinensis* were dried and manually ground to fine powder using a mortar and pestle, and lipids were extracted for 5 minutes with PE using an ultrasonic bath extraction method at room temperature. The ultrasonic frequency was 40 kHz, and the ratio of *C*. *sinensis* to PE was 1:4. The suspension was filtered, and the residue was re-extracted three times with three volumes of PE. The filtrates were combined, and the solvent was removed using a rotary evaporator (Heidolph, Schwabach) at 40°C under nitrogen to yield an extract of neutral lipids. Sample residues were re-extracted four times using three volumes of dichloromethane and methanol (1:1). The extraction solvent was removed, and the products were washed in distilled water to yield polar lipids.

Freshly prepared anhydrous THF (1 mL) and sodium methoxide (5%, 1 mL) were added to a solution of neutral lipids in 2 mL of anhydrous PE (2 mL of dichloromethane and methanol for polar lipids) in a penetrable screw-capped vial using a dry syringe. The mixture was subjected to vigorous shaking for 30 seconds and was left standing for ten minutes. The mixture was neutralized with acetic acid (5%, 1 mL) and was washed three times with distilled water. The PE layer, which contained fatty acid methyl esters (FAMEs), was dried using anhydrous sodium sulfate, filtered, and then preserved at 4°C.

#### Fatty acid analysis

FAME samples in PE were analyzed using an Agilent Technologies 6890 gas chromatograph (Agilent, Palo Alto, USA) equipped with a Gerstel MPS2 autosampler (Gerstel, Mülheim an der Ruhr, Germany), a Varian WCOT fused silica column (50 m length × 0.25 mm i.d., 0.25 m film thickness, CP-7419, Varian, Palo Alto, USA), and an Agilent 5973 mass spectrometer with the electron ionization mode (EI) generated at 70 eV (ion source at 230°C and transfer line at 280°C). The carrier gas, helium, was provided at a constant flow rate of 1.0 mL per minute. The following oven temperature program was used: initial oven temperature 100°C, held for two minutes; first program rate, 6°C/minute to 190°C and held for five minutes; second program rate, 20°C/minute to 260°C and held for five minutes. Blank runs were performed before sampling to prevent the carryover of analytes from the previous extraction. The FAME samples were diluted to approximately 50 ppm and were mixed with 10 ppm methylnonadecanoate (C_19:0_) as an internal standard. The injection volume was 1 L in the splitless mode. Individual FAMEs were identified by using mass spectrometry (MS) and also by comparison with the retention times of an external FAME standard (FAME Mix C4C24, Sigma-Aldrich, St. Louis, MO, USA). The FAME standard was diluted in acetone to produce concentrations of 10, 50, 100, 400, and 1,000 ppm. The standard curve was linear, and it was developed on the basis of the peak area of the total ion current (TIC) for the known concentration of the FAME standard. The absolute contents (mg of FAME per g of dry weight of sample) and the normalized relative contents (%) of fatty acids were calculated by comparisons with the standard curves.

### Statistical analysis

The experimental data were analyzed using IBM SPSS Statistics (Version 20, Microsoft, USA). The relative fatty acid abundances for each indoor *C*. *sinensis* sample were determined in triplicate. Their standard deviations were less than 0.3% and are expressed as the mean values (Tables 1 and 2). There were significant differences in the levels of C_18:3_, C_18:2_ and PUFAs between indoor and wild *C*. *sinensis* groups (*P* < 0.05, independent-samples *t* test).

**Table 1:** Relative abundances of fatty acids (%) in the neutral lipids from indoor *Cordyceps sinensis*.

Samples	C_14:0_	C_14:1_	C_15:0_	C_16:0_	C_16:1_	C_17:0_	C_17:1_	C_18:0_	C_18:1_	C_18:2_	C_18:3_	C_20:0_	C_20:1_	C_20:2_	C_22:0_	C_22:4_	C_24:0_	PUFAs
ICS 1	0.08	0.01	0.03	17.33	0.72	0.09	0.05	1.30	58.36	20.83	0.79	0.08	0.11	0.03	0.04	0.08	0.06	21.73
ICS 2	0.17	0.06	0.04	20.54	0.89	0.04	0.06	1.67	51.30	24.06	0.83	0.12	0.08	0.02	0.03	0.06	0.04	24.97
ICS 3	0.08	0.04	0.07	15.76	0.63	0.06	0.07	1.72	58.90	20.86	1.26	0.19	0.13	0.04	0.05	0.09	0.07	22.25
ICS 4	0.06	0.04	0.04	17.20	0.52	0.05	0.08	1.70	56.49	22.82	0.64	0.14	0.07	0.02	0.03	0.05	0.04	23.53
ICS 5	0.05	0.04	0.06	12.73	0.76	0.10	0.06	2.14	50.78	31.38	0.94	0.10	0.29	0.09	0.12	0.21	0.16	32.62
ICS 6	0.09	0.04	0.04	15.79	0.88	0.04	0.07	1.50	56.89	23.09	1.32	0.09	0.06	0.02	0.02	0.04	0.03	24.47
ICS 7	0.06	0.04	0.05	24.31	0.48	0.05	0.04	0.81	54.73	18.31	0.85	0.06	0.07	0.02	0.03	0.05	0.04	19.23
ICS 8	0.07	0.04	0.05	14.52	0.49	0.04	0.08	1.91	54.31	26.42	1.72	0.16	0.07	0.02	0.03	0.05	0.04	28.21
ICS 9	0.05	0.04	0.05	13.16	0.43	0.04	0.08	1.67	57.06	26.25	0.96	0.10	0.05	0.01	0.02	0.03	0.03	27.25
ICS 10	0.06	0.04	0.07	25.74	0.79	0.04	0.04	0.96	54.27	16.73	0.75	0.10	0.14	0.04	0.06	0.10	0.08	17.62
ICS 11	0.06	0.04	0.06	14.50	0.49	0.04	0.07	1.34	59.13	23.02	0.94	0.04	0.10	0.03	0.04	0.07	0.06	24.06
ICS 12	0.15	0.13	0.04	16.96	2.31	0.11	0.03	1.48	53.47	23.35	1.35	0.11	0.26	0.09	0.09	0.01	0.06	24.80
ICS 13	0.12	0.08	0.07	17.31	2.00	0.09	0.04	1.10	49.79	26.61	2.19	0.10	0.20	0.13	0.01	0.01	0.12	28.94
ICS mean	0.08	0.05	0.05	17.37	0.88	0.06	0.06	1.48	55.04	23.36	1.12	0.11	0.13	0.04	0.04	0.07	0.06	24.59
WCS mean	0.10	0.08	0.07	14.30	1.34	0.06	0.03	1.05	48.25	30.05	4.01	0.10	0.15	0.18	0.06	0.10	0.09	34.34

All data are the means of thrice determinations, and have the typical standard deviations ≤ 0.30% (SD_C16:0_ ≤ 0.10%; SD_C18:0_ ≤ 0.03%; SD_C18:1_ ≤ 0.27%; SD_C18:2_ ≤ 0.15%; SD_C18:3_ ≤ 0.09%; and the SDs of minor fatty acids ≤ 0.01%). PUFAs, poly unsaturated fatty acids; ICS 1–13, indoor *C*. *sinensis* samples; and WCS mean, the average value of wild *C*. *sinensis* produced on Sejila Mountain, Tibet [15].

**Table 2:** Relative abundances of fatty acids (%) in the polar lipids from indoor *Cordyceps sinensis*.

Samples	C_14:0_	C_14:1_	C_15:0_	C_16:0_	C_16:1_	C_17:0_	C_17:1_	C_18:0_	C_18:1_	C_18:2_	C_18:3_	C_20:0_	C_20:1_	C_20:2_	C_22:0_	C_22:4_	C_24:0_	PUFAs
ICS 1	0.06	0.04	0.17	15.65	0.55	0.12	0.37	5.35	27.30	48.61	1.10	0.26	0.11	0.15	0.04	0.07	0.06	49.93
ICS 2	0.05	0.04	0.15	15.52	0.45	0.06	0.23	4.67	30.58	47.03	0.71	0.27	0.06	0.08	0.02	0.05	0.12	47.87
ICS 3	0.07	0.04	0.18	15.28	0.35	0.07	0.41	6.51	31.72	43.01	1.15	0.43	0.20	0.24	0.07	0.13	0.02	44.53
ICS 4	0.04	0.04	0.11	15.21	0.19	0.05	0.31	5.48	29.61	47.74	0.68	0.12	0.10	0.12	0.04	0.07	0.04	48.61
ICS 5	0.08	0.04	0.30	16.50	0.82	0.06	0.51	6.80	22.01	51.47	0.94	0.31	0.04	0.04	0.01	0.03	0.04	52.48
ICS 6	0.04	0.04	0.15	13.41	0.27	0.07	0.46	6.22	23.44	54.41	0.98	0.31	0.06	0.07	0.02	0.04	0.08	55.50
ICS 7	0.05	0.04	0.14	20.75	0.37	0.04	0.18	4.19	24.12	48.15	1.41	0.33	0.07	0.08	0.03	0.05	0.03	49.69
ICS 8	0.08	0.04	0.22	14.32	0.30	0.05	0.41	7.00	25.76	49.65	1.19	0.42	0.15	0.18	0.05	0.11	0.04	51.13
ICS 9	0.04	0.04	0.23	12.41	0.32	0.03	0.57	6.14	24.29	54.95	0.60	0.18	0.06	0.07	0.02	0.04	0.08	55.66
ICS 10	0.04	0.04	0.11	19.57	0.49	0.05	0.58	3.61	32.29	41.48	1.24	0.25	0.07	0.08	0.02	0.05	0.07	42.85
ICS 11	0.04	0.04	0.16	14.00	0.28	0.31	0.32	5.02	30.15	47.87	0.94	0.19	0.30	0.20	0.08	0.07	0.07	49.08
ICS 12	0.06	0.03	0.13	12.99	0.95	0.45	0.09	4.78	25.06	53.97	1.05	0.07	0.24	0.11	0.00	0.01	0.01	55.14
ICS 13	0.11	0.06	0.19	17.88	1.77	0.26	0.33	3.76	28.59	43.60	2.31	0.19	0.00	0.56	0.10	0.19	0.10	46.66
ICS mean	0.06	0.04	0.17	15.65	0.55	0.12	0.37	5.35	27.30	48.61	1.10	0.26	0.11	0.15	0.04	0.07	0.06	49.93
WCS mean	0.08	0.11	0.11	14.85	0.98	0.27	0.03	6.54	13.05	56.30	3.33	0.54	0.87	1.33	0.79	0.30	0.67	61.25

All data are the means of thrice determinations, and have the typical standard deviations ≤ 0.30% (SD_C16:0_ ≤ 0.10%; SD_C18:0_ ≤ 0.03%; SD_C18:1_ ≤ 0.27%; SD_C18:2_ ≤ 0.15%; SD_C18:3_ ≤ 0.09%; and the SDs of minor fatty acids ≤ 0.01%). PUFAs, poly unsaturated fatty acids; ICS 113, indoor *C*. *sinensis* samples; and WCS mean, the average value of wild *C*. *sinensis* produced on Sejila Mountain, Tibet [15].

## Results

### Outcomes of the host larvae

**Fig 1:**
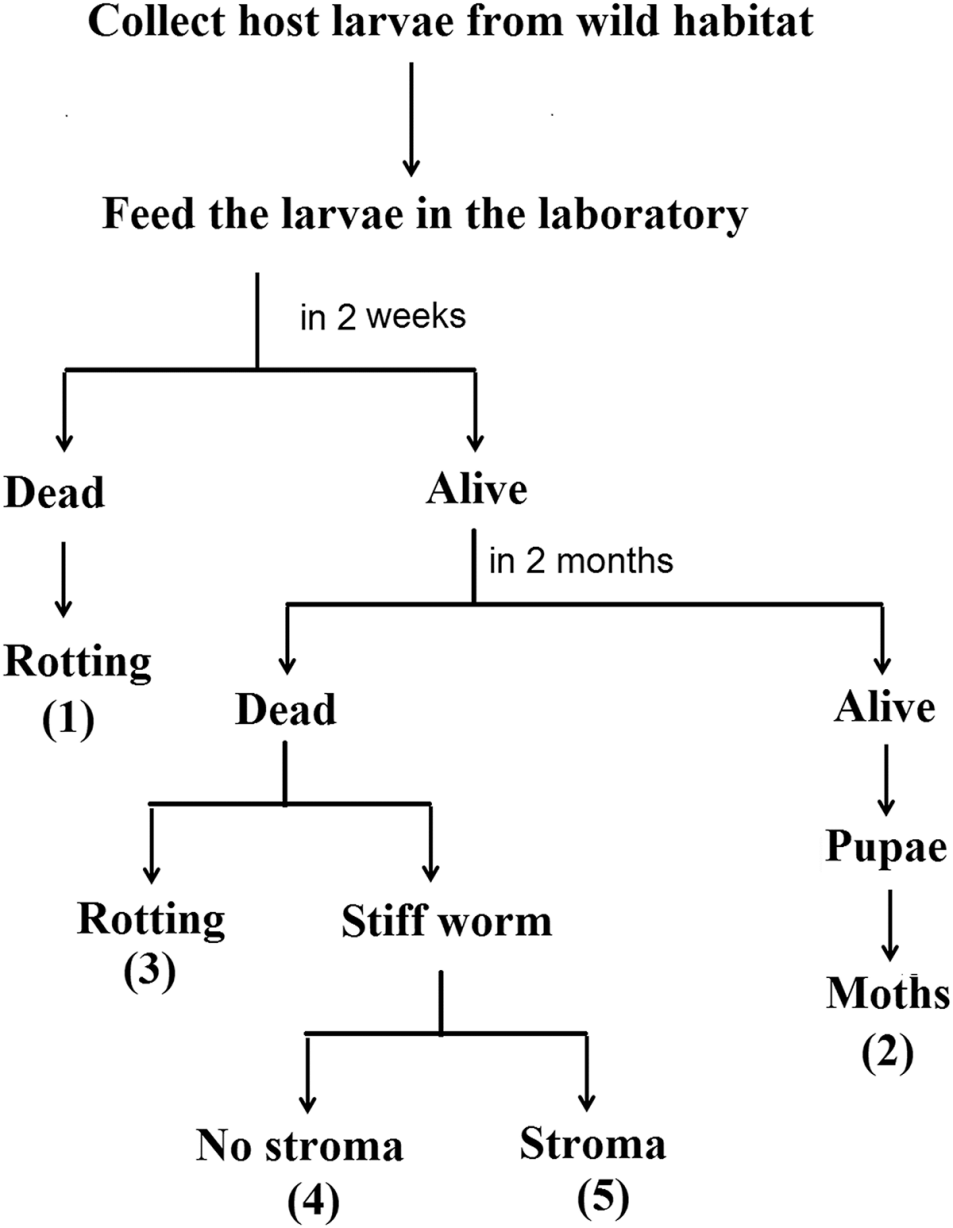
Outcomes of *Thitarodes* larvae in the laboratory.

After being brought to the laboratory, approximately 20% of the larvae gradually died over the course of two weeks (Fate 1 in Fig 1). The surviving larvae had the following four fates (Fates 2–5 in Fig 1):

Fate 2: approximately 25% of the surviving larvae continued to feed and eventually metamorphosed into pupae and moths; Fate 3: approximately 10% of the surviving larvae died and rotted, and their body fluids oozed out of the surface, giving off an unpleasant odor; Fate 4: approximately 30% of the surviving larvae died and became stiff worms with no stroma due to the infection of fungi such as *Metarrhizium*, *Paecilomyces* and *Beauveria* [23]; and Fate 5: approximately 35% of the surviving larvae died and became stiff worms because of the infection of dominant *H*. *sinensis* and eventually turned to *C*. *sinensis* with the stromata of *O*. *sinensis*, which was regarded as an indoor *C*. *sinensis* population in this study.

### Cultivation of indoor *C*. *sinensis* from the host larva

**Fig 2:**
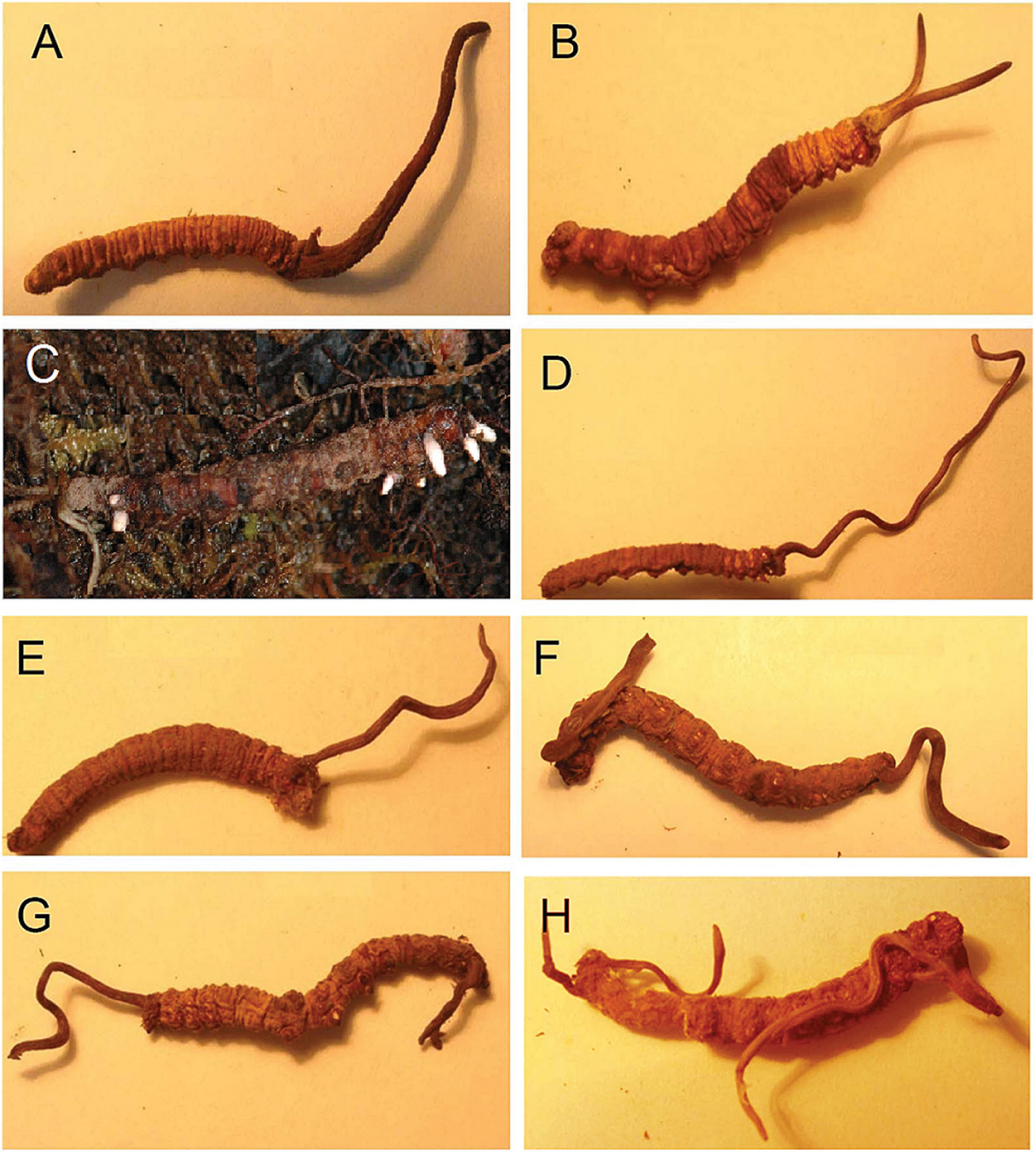
Comparison between the appearances of wild (A-B) and indoor (D-H) *Cordyceps sinensis*. (A) Single stroma in the head of a host *Thitarodes* larval cadaver; (B) Two stromata in the head; (C) Five and two initial white stromata in the head and tail, respectively; (D) Single stroma in the head; (E) Single stroma in the cephalothorax; (F-G) One stroma in the head and one stroma in the tail, respectively; and (H) One stroma and two stromata in the head and tail, respectively.

The development process from the host larva to *C*. *sinensis* was observed as follows. First, the infected larvae in the box became less vigorous and gradually transformed into yellowish, caterpillar-shaped, stiffened cadavers (stiff worms). In the following spring, stromata sprouted from the larval cadaver. The stromata, which were initially short and white (Fig 2C), gradually became longer and darker. By April, relatively long, light brown stromata had formed on the cadaver, which still clearly resembled a larva, including a head, neck, abdomen, and even multiple pairs of prolegs. The combination of stiff worm and stromata was regarded as indoor *C*. *sinensis* in this study.

The appearances of indoor *C*. *sinensis* are shown in Fig 2C to 2H. Most of the larvae had several slim stromata, which could occur at any position on a given larva. Indoor *C*. *sinensis* (Fig 2C to 2H) and wild *C*. *sinensis* (Fig 2A and 2B) exhibited clear differences in their appearances.

### Fatty acid composition of neutral and polar lipids from indoor *C*. *sinensis*

**Fig 3:**
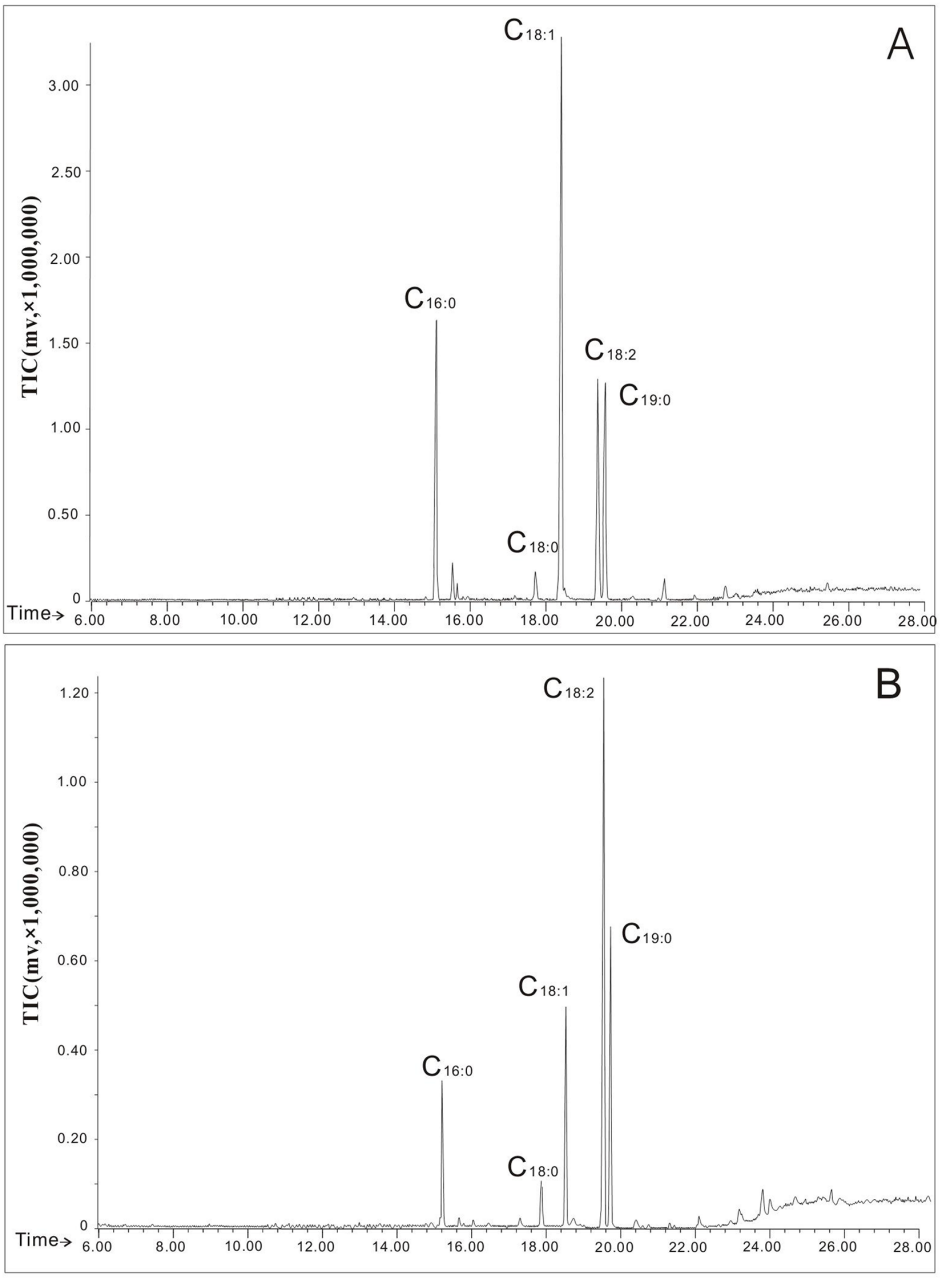
Total ion currents (TIC) of gas chromatography-mass spectrometry for the neutral (A) and polar (B) lipids from *Cordyceps sinensis*. C_16:0_, palmitic acid methyl ester; C_18:0_, stearic acid methyl ester; C_18:1_, oleic acid methyl ester; C_18:2_, linoleic acid; and C_19:0_, methylnonadecanoate as an internal standard.

Seventeen fatty acids were identified in the neutral and polar lipids from indoor *C*. *sinensis* using GC-MS (Fig 3), and their relative abundances and total polyunsaturated fatty acids (PUFAs) are presented in Tables 1 and 2, respectively. The major fatty acids among the neutral lipids were oleic acid (C_18:1,*cis*-9_, 49.79–59.13%), linoleic acid (C_18:2,*cis*-9,12_, 16.73–31.38%), and palmitic acid (C_16:0_, 12.73–25.74%); the minor fatty acids were palmitoleic acid (C_16:1,*cis*-9_, 0.43–2.31%), stearic acid (C_18:0_, 0.81–2.14%) and -linolenic acid (C_18:3_, 0.64–2.19%). The major fatty acids among the polar lipids were linoleic acid (C_18:2,*cis*-9,12_, 41.48–54.95%), oleic acid (C_18:1,*cis*-9_, 22.01–32.29%), palmitic acid (C_16:0_, 12.41–20.75%) and stearic acid (C_18:0_, 3.61–7.00%); the minor fatty acids were palmitoleic acid (C_16:1,*cis*-9_, 0.19–1.77%) and -linolenic acid (C_18:3_, 0.60 ~ 2.31%). In addition, myristic acid (C_14:0_), myristoleic acid (C_14:1,*cis*-9_), heptadecanoic acid (C_17:0_), heptadecenoic acid (C_17:1,*cis*-9_), eicosanoic acid (C_20:1,*cis*-11_), eicosadienoic acid (C_20:2,*cis*-11,14_), tetracosanoic acid (C_24:0_), pentadecanoic acid (C_15:0_), arachidic acid (C_20:0_), docosanoic acid (C_22:0_) and docosatetraenoic acid (C_22:4,*cis*-5,8,11,14_) were detected in minor amounts in both the neutral and polar lipids from indoor *C*. *sinensis*.

**Fig 4:**
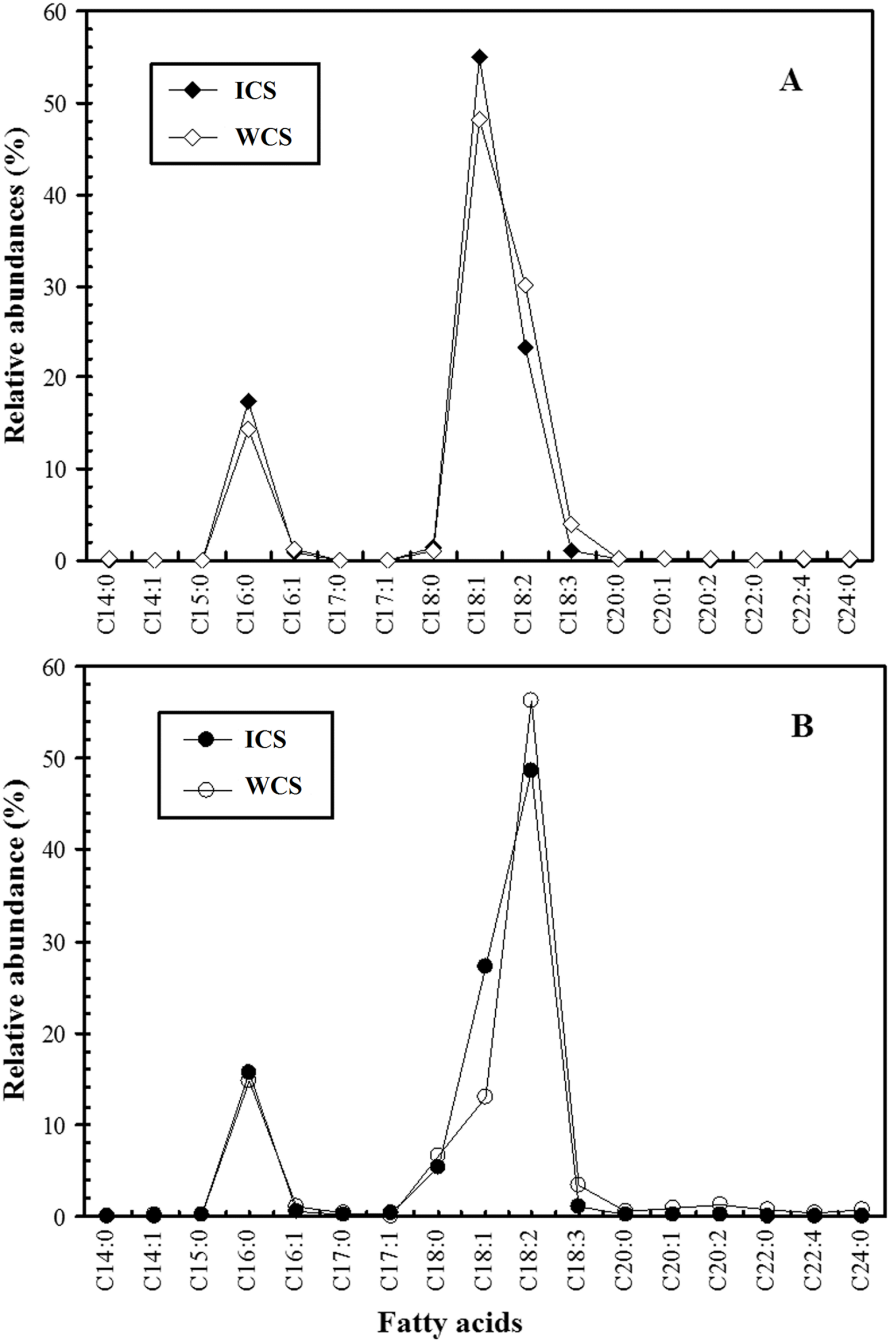
Fatty acid patterns showing the fatty acid profiles of neutral (A) and polar (B) lipids from *Cordyceps sinensis*. ICS, indoor *C*. *sinensis*; WCS, wild *C*. *sinensis*.

**Fig 5:**
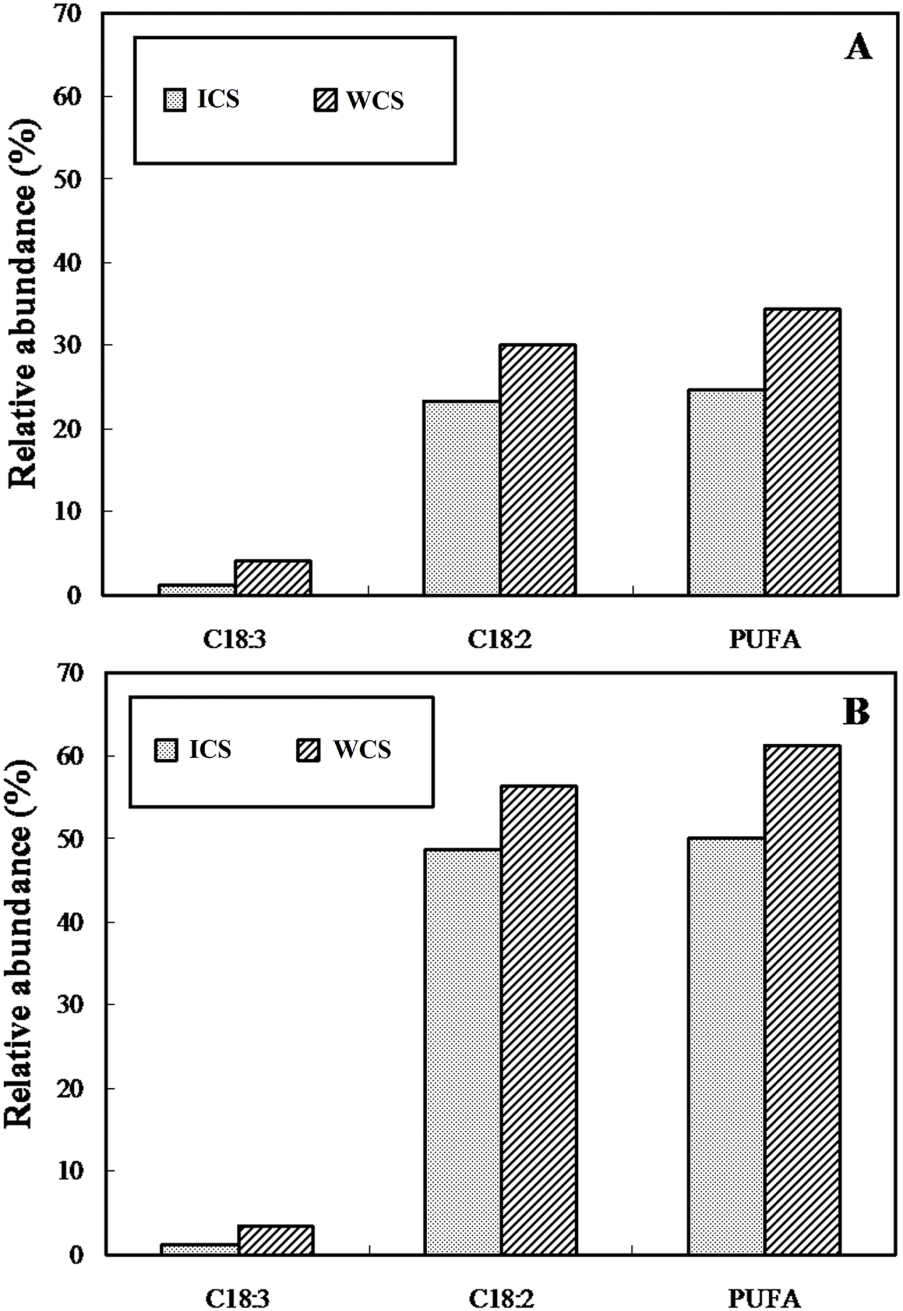
Histogram showing the relative abundances of C_18:3_, C_18:2_ and total PUFAs in neutral (A) and polar (B) lipids from *Cordyceps sinensis*. ICS, indoor *C*. *sinensis*; WCS, *C*. *sinensis*.

Based on the data in Tables 1 and 2, the data points are joined by lines in Fig 4 to illustrate the specific fatty acid profiles. Fig 4 shows that indoor and wild *C*. *sinensis* possessed similar fatty acid profiles in neutral and polar lipids, with two specific peaks at C_16:0_ and C_18:1_ in the neutral lipids and two specific peaks at C_16:0_ and C_18:2_ in the polar lipids. Furthermore, the levels of C_18:2_ and C_18:3_ in the neutral (30.05% and 4.01%) and polar lipids (56.30% and 3.33%) from wild *C*. *sinensis* [15] were higher in the wild *C*. *sinensis* than in the indoor *C*. *sinensis* (23.26% and 1.12% in neutral lipids, and 48.61% and 1.10% in polar lipids, respectively). The distinct differences (*P* < 0.05) in the levels of C_18:2_, C_18:3_ and PUFAs between indoor (PUFAs: 24.59% in neutral and 49.93% in polar lipids) and wild *C*. *sinensis* (PUFAs: 34.34% in neutral and 61.25% in polar lipids) are illustrated in Fig 5, showing that three fatty acids among the neutral and polar lipids were much lower in abundance in indoor *C*. *sinensis* than in wild *C*. *sinensis* [15].

## Discussion

### Occurrence of *O*. *sinensis* (stroma) from the infected host larva

The incubation of *O*. *sinensis* is a complicated process consisting of three indispensable stages: infection, parasitism and saprophytism by *H*. *sinensis* [14]. When the wild host larvae were cultivated in the laboratory without any intentional artificial inoculation, a subset of them still eventually became *C*. *sinensis*. Thus, an immediate question follows: How were the larvae infected? The omnivorous larvae with their troglodytic life in soils prefer to eat the tender roots of plants and even the soil humus [20]. In spring and summer, the erupted spores of *H*. *sinensis* disperse in the topsoil and develop into infective conidia, which gradually infiltrate into the ground with the rainfall. The larvae might be infected by *H*. *sinensis* from habitat soils through the body-surface contact and digestive tract before collection. Logically, all of the larvae had an equal opportunity to be infected by *H*. *sinensis*. However, our observations under the same culture conditions and without any artificial inoculation suggested that the fates of larvae manifested a distinct difference, *i*.*e*., only *ca*. 35% of the larvae could eventually transform into *C*. *sinensis* (Fate 5 in Fig 1). Previous studies have suggested that wild larvae showed a probability of less than 1/1000 of being infected by *H*. *sinensis* [22], and this likelihood is evidently related to the amino acids and minor elements present in the larvae [24]. Obviously, certain intrinsic characteristics of larvae are rather than environmental factors affect their destiny. Consequently, we consider that there is a competitive relationship in the immune system of larvae between *H*. *sinensis* and other microorganisms. If *H*. *sinensis* wins this competition, the infected larvae may enter the saprophyte stage and ultimately develop to become *O*. *sinensis*. Because the optimal growth temperatures of *H*. *sinensis* (1518°C) [25] are lower than those of other microorganisms (2530°C) [26,27], we consider that the low-temperature incubation may be an effective way to promote the infection of the larvae by *H*. *sinensis* and inhibit infection by other microorganisms. Our above inference is consistent with the previous report that the stroma of *O*. *sinensis* could geminate only after incubation at low temperature (near 0°C) [28].

### Influence of growth temperature on lipid synthesis by *O*. *sinensis*

Temperature is also an important factor affecting the fatty acid compositions of organisms. In our previous study, different groups of host larvae were reared for 30 days at −3°C, 0°C, 4°C, 8°C, 12°C and 15°C, respectively, and their fatty acid compositions were significantly affected by low temperature [29], *i*.*e*., the unsaturated fatty acids increased and saturated fatty acids decreased with the reduction in temperature. Previous studies have also suggested that alternating membrane lipid composition is one strategy utilized by fungi to adapt to low or high temperature stress [30]. Freeze damage to cells occurs when membrane lipids transition from liquid crystalline to a gel phase at low temperatures. *O*. *sinensis* may respond to low temperatures by altering its lipid composition, increasing the proportion of unsaturated lipids and the unsaturation indices to sustain cell function [31]. Our previous study demonstrated that the contents of PUFAs in wild *C*. *sinensis* from Sejila Mountain were higher than those in *C*. *sinensis* from other habitats, which might be closely related to the long-term maintenance of thicker permafrost due to the relative high humidity on Sejila Mountain [15]. In this study, the mean indoor temperature was higher than that outside the laboratory. The relative abundances of C_18:2_, C_18:3_ and total PUFAs were much lower in the indoor samples than in the wild samples. Differences (*P* < 0.05, independent-samples *t* test) in the PUFAs between indoor (24.69% in neutral and 49.93% in polar lipids) and wild *C*. *sinensis* (34.34% in neutral and 61.25% in polar lipids) [15] may be attributable to their different growth temperatures. *O*. *sinensis* and its host larvae are found on Sejila Mountain with an elevation of 4,200 m. The annual average temperature is −0.7°C in this habitat, and the monthly mean temperature ranges from −14.0°C (January) to 15.5°C (August). The minimum and maximum temperatures are −31.6°C and 24.0°C, respectively [32]. Indoor *C*. *sinensis* was cultivated at the same altitude as the Sejila Mountain habitat, but the average temperatures in the laboratory were higher than outside. Therefore, we infer that *O*. *sinensis* requires a higher proportion of PUFAs to maintain its cell membrane fluidity and physiological functions in response to chilling outside temperatures in the wild. Therefore, temperature, especially temperature change, is one key environmental factor that affects the growth of *O*. *sinensis* and should be considered in its large-scale indoor cultivation.

### Environmental factors affecting the appearance of *O*. *sinensis*

#### Soil pressure

In the wild, the host *T*. larvae generally inhabit soils at depths of 1535 cm, and they do not emerge from the soil until after pupating [33]. After becoming infected by the fungus *H*. *sinensis* in late autumn, the larvae prefer to turn their heads up nearer the soil surface for more favorable respiration [7]. In the subsequent winter, the infected larvae die and turn into “stiff worms”. In the following spring and summer seasons, the stroma germinates and sprouts from the head of the larva. Clearly, this position is also beneficial for their germination (Fig 2A) [7,13]. Accordingly, the majority of wild *C*. *sinensis* had a single, plump stroma that germinated vertically from the larval head (Fig 2A) [7,13]; only a small number possessed multiple stromata [34] (Fig 2B).

In our study, wild host *T*. larvae were collected and delivered to the laboratory for feeding. Just as described above for the wild larvae, in the coming winter, some of them died and gradually turn into muscardine cadavers (“stiff worms”). To more clearly observe the occurrence of *O*. *sinensis*, each muscardine cadaver was incubated in a separate box. The stroma sprouted and grew freely because of the absence of soil pressure. Therefore, *C*. *sinensis* with multiple stromata of *O*. *sinensis* predominated (Fig 2C); multiple lean and bent stromata could germinate at any position of stiffened larvae, with wild and indoor fungi exhibiting distinctly different appearances (Fig 2D to 2H). Therefore, we infer that the soil pressure may control the sprouting positions of *O*. *sinensis* stromata.

#### Light intensity

In the wild, *O*. *sinensis* began to germinate stroma in late March, and the stromata grew upwards quickly at a rate of approximately 3 mm per day below the soil surface. Exposure to adequate sunlight caused the growth of the stromata to suddenly slow to 1.5 mm per day when they emerged from the soil surface [35]. Long-term observations showed a distinct negative correlation between average stroma length and light intensity. Moreover, stromal growth exhibited evident negative phototaxis, *i*.*e*., if the sunlight was sheltered in one side, stroma would turn away from the sunlight in their early growth stage [35]. Therefore, in the wild, severe sunlight stress not only inhibited stromal overgrowth but also promoted upward growth, sufficient size, and strength after stromal emergence from the ground (Fig 2A).

In the laboratory, the muscardine cadaver (“stiff worm”) was cultivated in an incubator with a cover for maintaining stable temperature and humidity. The light deficiency (10–30 lx) in the incubator might promote the overgrowth of stromata, which become long and slim (Fig 2D to 2H). Moreover, no directional light could induce stromata to grow in any direction or to curl (Fig 2D to 2H). The overgrowth of stromata caused the caterpillar-shaped sclerotium to become hollow and decayed and finally to lose its medicinal value. Therefore, the light intensity will likely be an important environmental factor in controlling the growth of *O*. *sinensis* and preventing negative effects on its medicinal efficacy.

#### Temperature

Temperature is also an important factor affecting the development of *O*. *sinensis*. Tu et al. reported that the muscardine cadaver could not geminate a stroma in the constant-temperature cultivation at 12°C, and the stroma could geminate only if it underwent a period of low temperature (near 0°C) incubation [28]. Our observations indicated that multiple stromata sprouted from the muscardine cadaver in the range of −4°C to 4°C, which was consistent with the previously reported result [28].

In conclusion, the observations of the life cycle from the wild host larva to indoor-cultivated *C*. *sinensis* at a high-altitude laboratory on Sejila Mountain are reported for the first time. The morphological characters and fatty acid compositions of neutral/polar lipids from indoor-cultivated *C*. *sinensis* indicate that environmental factors, especially temperature, soil pressure and light intensity, strongly affect the growth of *C*. *sinensis*. Our new results may provide important evidence for improving the techniques for the large-scale artificial cultivation of *C*. *sinensis*.
